# Impact of Birthing Room Design on Maternal Childbirth Experience: Results From the Room4Birth Randomized Trial

**DOI:** 10.1177/19375867221124232

**Published:** 2022-10-14

**Authors:** Lisa Goldkuhl, Hanna Gyllensten, Cecily Begley, Christina Nilsson, Helle Wijk, Göran Lindahl, Kerstin Uvnäs-Moberg, Marie Berg

**Affiliations:** 1 Institute of Health and Care Sciences, Sahlgrenska Academy, University of Gothenburg, Sweden; 2 Department of Obstetrics and Gynaecology, Sahlgrenska University Hospital, Gothenburg, Sweden; 3 University of Gothenburg Centre for Person-Centred Care (GPCC), Sahlgrenska Academy, University of Gothenburg, Sweden; 4 School of Nursing and Midwifery, Trinity College Dublin, The University of Dublin, Ireland; 5 Munkebäck Antenatal Clinic, Region Västra Götaland, Gothenburg, Sweden; 6 Department of Quality Assurance and Patient Safety, Sahlgrenska University Hospital, Gothenburg, Sweden; 7 Centre for Healthcare Architecture, CVA, Chalmers University of Technology, Gothenburg, Sweden; 8 Department of Architecture and Civil Engineering, Building Design, Chalmers University of Technology, Gothenburg, Sweden; 9 University of Agriculture (SLU), Uppsala University, Sweden; 10 Faculty of Medicine and Community Health, Evangelical University in Africa, Bukavu, D. R. Congo

**Keywords:** childbirth experience, maternal health, healthcare environment, physical design, birthing room design, randomized controlled trial, longitudinal studies

## Abstract

**Objective:**

To study the effect of the birthing room design on nulliparous women’s childbirth experience up to 1 year after birth.

**Background:**

Although it is known that the birth environment can support or hinder birth processes, the impact of the birthing room design on maternal childbirth experience over time is insufficiently studied.

**Methods:**

The Room4Birth randomized controlled trial was conducted at a labor ward in Sweden. Nulliparous women in active stage of spontaneous labor were randomized (*n* = 406) to either a regular birthing room (*n* = 202) or a new birthing room designed with more person-centered considerations (*n* = 204). Childbirth experiences were measured 2 hr, 3 months, and 12 months after birth by using a Visual Analogue Scale of Overall Childbirth Experience (VAS-OCE), the Fear of Birth Scale (FOBS), and the Childbirth Experience Questionnaire (CEQ2).

**Results:**

Women randomized to the new room had a more positive childbirth experience reported on the VAS-OCE 3 months (*p* = .002) and 12 months (*p* = .021) after birth compared to women randomized to a regular room. Women in the new room also scored higher in the total CEQ2 score (*p* = .039) and within the CEQ2 subdomain *own capacity* after 3 months (*p* = .028). The remaining CEQ2 domains and the FOBS scores did not differ between the groups.

**Conclusions:**

These findings show that a birthing room offering more possibilities to change features and functions in the room according to personal needs and requirements, positively affects the childbirth experience of nulliparous women 3 and 12 months after they have given birth.

The experience of childbirth is of significant meaning for women and their families, both short and long term (Bossano et al., 2017). In the World Health Organization (2018) recommendations for intrapartum care, it has been emphasized that focus should be expanded to not only ensure the survival of women and babies following childbirth but also to include the importance of a positive and healthy experience. This is essential from a mental health perspective, since it is known that childbirth satisfaction can empower and increase a person’s sense of self and positively impact the mother–infant attachment (Weisman et al., 2010). A positive childbirth experience is associated with having an uncomplicated birth, receiving individualized support from trusting care providers and midwifery continuity of care (Sandall et al., 2016). It is also associated with having a sense of control and involvement in decision-making during labor (Townsend et al., 2020). In contrast, dissatisfaction with childbirth can result in postpartum depression and post-traumatic stress disorder, which can have a negative impact on breastfeeding and the mother–infant bonding (Cook et al., 2018; Leinweber et al., 2022). Negative childbirth experiences can also have societal and economic effects, since women may request to delay or avoid giving birth again due to intense fear after a traumatic experience. They may also request for a future elective caesarean birth, which is associated with increased risks of adverse outcomes for women and neonates (Sandall et al., 2018).

Contextual and psychosocial elements within the birth environment significantly influence childbirth experiences and physiological birth processes (Olza et al., 2020). For instance, a perceived safe environment is associated with childbirth satisfaction and enables production and release of the labor hormone oxytocin. Oxytocin does not only induce and stimulate labor contractions but also reduces pain and stress levels and positively influences emotional well-being. These effects are induced via oxytocinergic nervous connections in the brain, which are triggered along with the oxytocin release into the circulation (Uvnas-Moberg et al., 2019). In addition, the oxytocin-induced effects that occur during birth may have long-lasting consequences. Since oxytocin has an amnesic effect, this may reduce the memory of a negative childbirth experience (Olza et al., 2020; Uvnas-Moberg et al., 2019). Personalized, calming, and supportive birth environments, thereby, need to be offered to women for their physiological process of labor to function optimally and to reduce the risk of birth complications, such as prolonged labor (Olza et al., 2020). This is particularly necessary since there is evidence showing that intrapartum complications and medical interventions, such as oxytocin augmentation, instrumental vaginal birth, and emergency caesarean birth, are factors contributing to childbirth dissatisfaction (Hosseini Tabaghdehi et al., 2020).

***Contextual and psychosocial elements within the birth environment significantly influence childbirth experiences and physiological birth processes***.

Having less clinical-like indoor hospital environments with controllable sensory stimuli, aromas, sounds, lights, comfortable furniture, and views of nature are beneficial for the health of persons admitted to hospitals (Ulrich et al., 2008). For birth spaces, evaluating the effect of the physical environment on birth outcomes has garnered recent interest, but results are inconclusive (Nilsson et al., 2020; Setola et al., 2019). It has been shown that specially designed hospital birthing rooms offering a sense of familiarity and different multisensory attributes can reduce women’s requirements of epidural analgesia during labor (Goldkuhl, Gyllensten et al., 2022) and lower caesarean birth rates (Wronding et al., 2019). However, these birth environments are in many trials implemented in settings with several other confounding factors related to the organizational model of care, such as continuity of carer, which is known to positively affect birth outcomes (Hodnett et al., 2012; Lorentzen et al., 2021).

There is also a lack of research concerning the effect of the built environment on women’s childbirth experience over time (Nilsson et al., 2020). Therefore, this study aimed to investigate the impact of the birthing room design on the childbirth experience of nulliparous women. We hypothesized that when the physical design supports physiological birth by offering women enhanced freedom of changing features and functions in the birthing room, the likelihood of a positive childbirth experience will increase up to 12 months after they have given birth.

***We hypothesized that when the physical design supports physiological birth by offering women enhanced freedom of changing features and functions in the birthing room, the likelihood of a positive childbirth experience will increase***.

## Method

### Study Design and Setting

This study reports women’s childbirth experiences 2 hr, 3 months, and 12 months after participation in the Room4Birth randomized controlled superiority trial (RCT) in Sweden (Goldkuhl, Gyllensten et al., 2022). Women were randomized to give birth in either (i) a regular hospital birthing room (regular room, control group) or (ii) a refurbished room designed with more person-centered considerations (new room, intervention group). The trial was conducted in accordance with the Declaration of Helsinki (World Medical Association, 2013), registered at ClinicalTrial.gov (NCT03948815), and ethically approved by the Regional Ethics Board (Dnr:478-18). The study procedures for the RCT followed the CONSORT guidelines and are described in detail in a study protocol (Berg et al., 2019).

**Figure 1.**
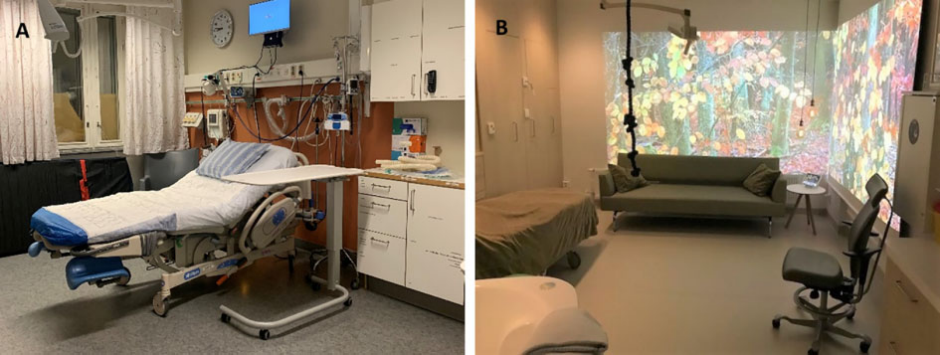
Photos of (A) one of the regular birthing rooms and (B) the new birthing room.

**Figure 2.**
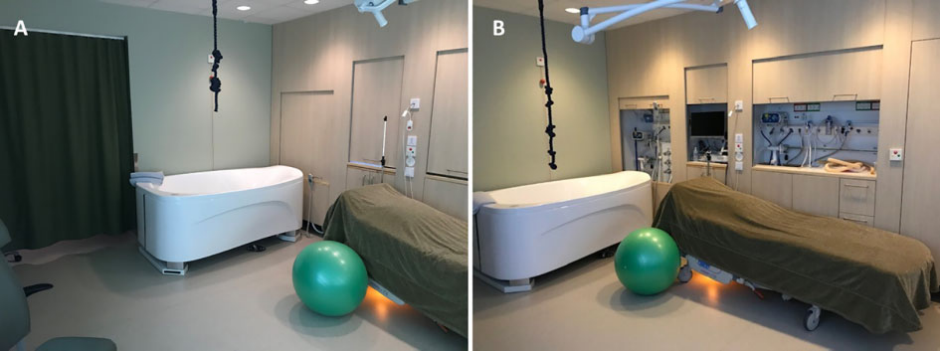
New birthing room where the medico-technical devices were hidden behind wood-paneled walls (A) that could be rolled up if required (B).

Randomization was undertaken between January 2019 and October 2020 at a labor ward located in western Sweden and with an annual birth rate of around 4,000 births (Swedish Pregnancy Register, 2022). All randomized participants had access to the same level of professional support and labor analgesia regardless of allocated group. The birthing rooms in the study labor ward were also equally equipped with medico-technical devices. What differed between the two randomized groups was that the new room provided users with more opportunities to adjust features and functions in the room compared with in the seven, similarly designed regular birthing rooms (Figure 1). Women and companions in the new room could control different aspects of the room, such as the degree of the dimmable light and the position of the bed. The women also had access to a large bathtub and more options for upright birth position. Additionally, the new room was designed to induce the feelings of calmness and familiarity through a less clinical-like decor. For instance, the medico-technical devices in the room were covered by sliding wooden panels that could be raised if preferred or needed (Figure 2). Unlike in the regular rooms, users of the new room had access to programmed nature scenes displayed on two of the walls, combined with either classical music or nature sounds. There was also an entrance hall with a green-colored curtain that protected the new room from being seen from the hospital corridor outside.

### Sample and Recruitment

Eligible participants for randomization were women aged 18 or more, classified as Robson 1 (Robson et al., 2015); nulliparous with a single, live, cephalic fetus at term and with spontaneous onset of labor. Participants also needed to be in active stage of labor as defined in Sweden by the time of recruitment (two of the following three criteria fulfilled at labor ward admission: two or three labor contractions in 10 min, spontaneous rupture of membranes, and cervix dilated >3 cm or effaced and open >1 cm). Furthermore, only women who understood either Swedish, English, Arabic, or Somali or had access to an interpreter could be recruited. Women in induced or latent phase of labor or with a planned caesarean birth were excluded.

Participant recruitment was carried out by the midwives and assistant nurses at the labor ward. Eligible women were asked to participate after labor ward arrival and confirmed active stage of labor, if both a regular room and the new room were vacant. All women gave signed, written consent to participate, and the time interval from labor ward arrival to randomization was set to be as short as possible to ensure an early allocation to the randomized room. The randomly computer-generated allocation list was managed by an independent agency. Information about the allocated room was printed in sealed, opaque envelopes sequentially placed in a study box at the labor ward. The recruiting care provider was not aware of the randomization sequence, and the independent agency ensured that the allocation list was followed. The women were provided with a four-digit ID code, which was printed on the sealed envelope.

### Data Collection and Outcome Measures

Participants’ demographic data were collected from obstetric records and through a self-reported, digital questionnaire 2 hr after birth (*Follow-Up 1*), while they were still in the birthing room. The questionnaire included a Visual Analogue Scale of Overall Childbirth Experience (VAS-OCE 1-10) and a modified Fear of Birth Scale (FOBS 0-100) (Haines et al., 2011; Hildingsson et al., 2017) and was available in Swedish, English, Arabic, and Somali (Supplementary Information 1). Data from *Follow-Up 1* have been reported in an earlier publication (Goldkuhl, Gyllensten et al., 2022) but were also used in this study to measure the difference in childbirth experience over time.

**Figure 3.**
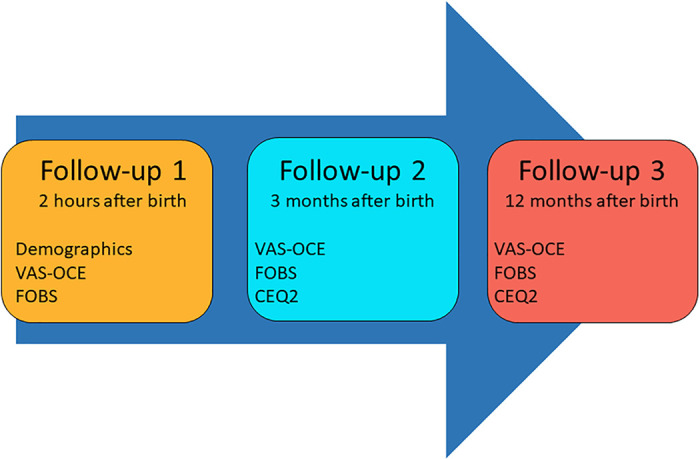
Overview of data collection. Participants’ self-reported experiences on a Visual Analogue Scale of Overall Childbirth Experience (VAS-OCE), Fear of Birth Scale (FOBS), and Childbirth Experience Questionnaire version 2 (CEQ2).

After 3 (*Follow-Up 2*) and 12 months (*Follow-Up 3*), all participants were asked to complete another online questionnaire (Supplementary Information 2) sent to their email address with two reminders to nonresponders. These questionnaires included VAS-OCE, FOBS, and the Childbirth Experience Questionnaire version 2 (CEQ2) (Dencker et al., 2020; Walker et al., 2020). A data collection overview is shown in Figure 3. The time intervals of follow-up were chosen based on research, showing that the experience of childbirth changes over time (Lundgren, 2005; Waldenström, 2003, 2004). It is, however, difficult to determine the most reliable time of measuring childbirth experience since it is a multidimensional and complex phenomenon. Reflections from a more long-term perspective may, nonetheless, be favorable since they tend to be more nuanced (Lundgren, 2005).

Assessing overall childbirth experience by using a VAS (continuous data) ranging from 1 to 10, where 10 is most positive, is routinely used in Sweden (Swedish Pregnancy Register, 2022). In the follow-up questionnaires 2 and 3, the participants self-reported their overall experience on a 100-mm linear analogue scale and not 1–10 as in *Follow-Up 1*, since it was in congruence with the linear scales in the other instruments (CEQ2 and FOBS). Since VAS most often is used to measure pain, we chose to name the variable VAS-OCE to clarify that we measure overall childbirth experience.

To assess fear during birth and of a potential future birth, a modified FOBS was used. In its original form (Haines et al., 2011; Hildingsson et al., 2017), participants are asked to put a mark on two 100-mm linear analogue scales during pregnancy, which answers the question: *How do you feel right now about the approaching birth?* Answers are rated from calm to worried (0–100) and from no fear to strong fear (0–100), and the scales are summed and averaged to calculate a score. Since participants for this study answered the questionnaire after birth, we modified the FOBS to report the question retrospectively and prospectively: *How do you rate worry and fear when you think about your completed labor and birth?* and *How do you rate worry and fear when you think about giving birth again?.* Higher scores in the FOBS represent stronger childbirth fear. As suggested by the creators, a cutoff point of 60 and above was used to define the fear of childbirth (Hildingsson et al., 2017).

The CEQ2 (Dencker et al., 2020; Walker et al., 2020) is a validated instrument containing 22 questions in four different domains (*own capacity*, *perceived safety*, *professional support*, *and participation*; Supplementary Information 2) concerning the childbirth experience. For 19 of the CEQ2 items, the response format is a 4-point Likert-type scale ranging from 1 = *totally disagree*, 2 = *mostly disagree*, 3 = *mostly agree* to 4 = *totally agree*. For the remaining three items (perceived pain, control, and sense of security), answers are assessed on a linear analogue scale (0–100), which are subsequently transformed to categorical variables: 1 = 0–40, 2 = 41–60, 3 = 61–80, and 4 = 81–100. The items produce mean scores in the four domains as well as a total CEQ2 score. Higher scores in the CEQ2 reflect more positive experiences. The few women who chose to answer the self-reported questionnaire at 2 hr in Arabic (two women in the new room and none in the regular room) or Somali (one woman in the new room and none in the regular room) received the questionnaires at 3 and 12 months in Swedish, since there were no validated translations for FOBS and CEQ2 in these languages.

### Statistical Analysis

To compare differences between the two randomized groups, χ^2^ test was used for nonordered categorical variables and Fisher’s exact tests for dichotomous variables. For group comparisons of the continuous variables included in the instruments (VAS-OCE, FOBS, and CEQ2), Mann Whitney *U* test was used due to nonnormally distributed data. We also estimated effect sizes *r*, as proposed by Cohen (1988) to examine the degree of variance between the groups, where *r* > .1 is considered a small effect, *r* > .3 medium effect, and *r* > .5 large effect (Fritz et al., 2012). Results for categorical variables are presented with *n* (%), and for continuous outcome variables as mean (standard deviation), median, quartile 1 and 3, and 95% confidence intervals for mean. Cronbach’s α was used to evaluate the internal consistency reliability of the domains included in the CEQ2 questionnaire.

A linear mixed effects model was used to describe the group differences in childbirth experience over the three time points. This regression model was chosen since it can provide information about the women’s individual change in childbirth experience over time but is also flexible since it has the capability to handle missing observations in the repeated measures data (Griffiths et al., 2017). The model examined the fixed effects of time and randomized group as well as the random effect of time on each of the dependent variables represented in VAS-OCE, FOBS, and CEQ2. A subsequent analysis controlling for the use of epidural analgesia and oxytocin augmentation during labor were conducted, due to the detected group differences in these variables and since these are factors known to affect the childbirth experience (Hildingsson et al., 2021). The analyses were performed using SPSS v.28 (SPSS IBM Statistics) for the comparisons between the two randomized groups and Stata (17.0, StataCorp LLC, College Station, TX) for the mixed effects model. All analyses were conducted according to the intention-to-treat methodology, and the significance tests were two-sided and had a significance level set at 5%.

## Results

**Figure 4.**
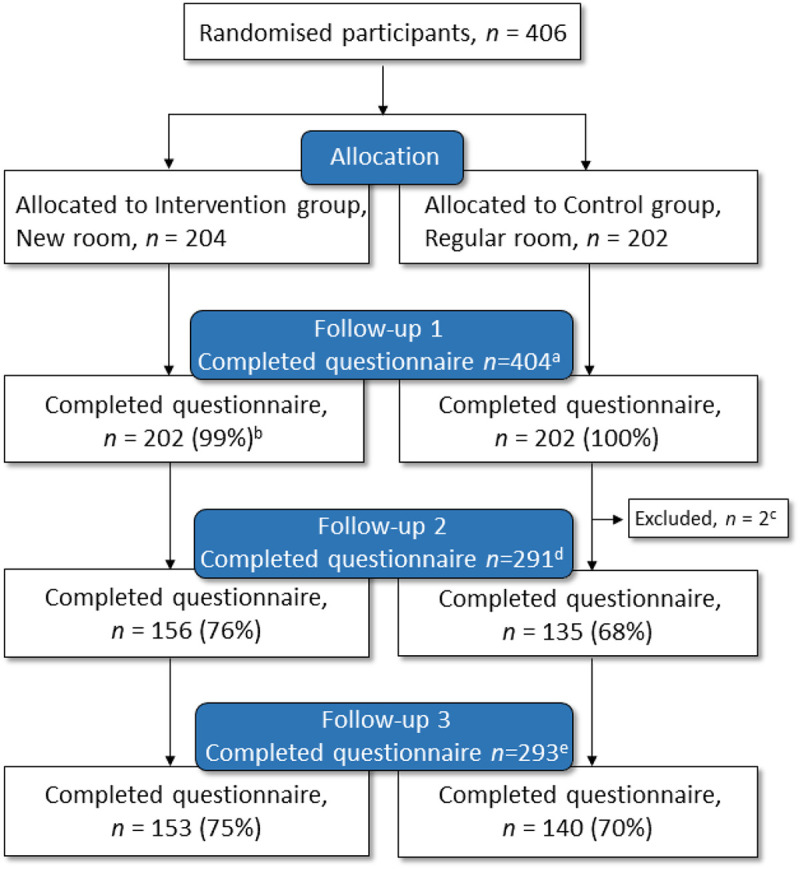
CONSORT flow chart of randomization and follow-up. ^a^ 99.5% completed the questionnaire 2 hr after birth.^b^ Two lost to follow-up due to study routine error. ^c^ No email address reported by the study participant. ^d^ 72% completed the questionnaire 3 months after birth. ^e^ 73% completed the questionnaire 12 months after birth.

The study sample consisted of 406 women, where 204 were randomized to the new room and 202 to the regular room (Figure 4). Response rates for the follow-up questionnaires were 99.5% (99% in the new room and 100% in the regular room) at 2 hr, 72% (76% in the new room and 68% in the regular room) at 3 months, and 73% (75% in the new room and 70% in the regular room) at 12 months. There were no incomplete questionnaires since the digital format indicated if there were any missing answers before submission. None of the Arabic- or Somali-speaking participants (*n* = 3) answered *Follow-Up 2*, but two of them answered *Follow-Up 3* in Swedish. Of the total study sample, 397 participants were included in the per protocol population. Among the nine not included according to protocol, eight were recruited in latent phase of labor and one was erroneously allocated to neither the regular room nor the new room. The per protocol population included 396 responders to *Follow-Up 1*, 285 responders to *Follow-Up 2*, and 286 responders to *Follow-Up 3*.

The demographic characteristics of the responders to the follow-up questionnaires did not differ between the randomized groups (Table 1). Responders to the 3- and 12-month questionnaires were more likely to be born in Europe (91%, respectively) compared with nonresponders (77% of all 3-month nonresponders and 76% of all 12-month nonresponders). Women randomized to the new room used the bath for water immersion to a significantly higher degree and required epidural analgesia to a significantly lower degree than women in the regular room, which has been reported previously (Goldkuhl, Gyllensten et al., 2022).

**Table 1.** Participants’ Demographic Information.

Variables	2-Hr Follow-Up		3-Month Follow-Up		12-Month Follow-Up	
	New Room (*n* = 202)	Regular Room (*n* = 202)	New Room (*n* = 156)	Regular Room (*n* = 135)	New Room (*n* = 153)	Regular Room (*n* = 140)
Country of birth, *n* (%)						
Sweden	149 (73.8)	155 (76.7)	123 (78.8)	108 (80.0)	122 (79.7)	112 (80.0)
Other Nordic countries	7 (3.5)	2 (1.0)	7 (4.5)	2 (1.5)	7 (4.6)	1 (.7)
Other European countries	22 (10.9)	18 (8.9)	14 (9.0)	11 (8.1)	11 (7.2)	15 (10.7)
Countries outside Europe	24 (11.9)	27 (13.4)	12 (7.7)	14 (10.4)	13 (8.5)	12 (8.6)
Maternal age (years)						
Mean (*SD*)	29.6 (4.5)	30.2 (3.9)	29.9 (4.3)	30.4 (3.9)	30 (4.1)	30.6 (3.8)
Median (IQR)	29 (27, 32.3)	30 (28, 32)	30 (27, 33)	30 (28, 32)	30 (27, 32)	31 (28, 33)
Educational level, *n* (%)						
Compulsory school	5 (2.5)	3 (1.5)	2 (1.3)	0 (0)	2 (1.3)	1 (.7)
High school	48 (23.8)	35 (17.3)	35 (22.4)	20 (14.8)	31 (20.3)	19 (13.6)
University or college	149 (73.8)	164 (81.2)	119 (76.3)	115 (85.2)	120 (78.4)	120 (85.7)
Family situation, *n* (%)						
Cohabitation with partner	191 (94.6)	195 (96.5)	149 (95.5)	133 (98.5)	145 (94.8)	137 (97.9)
Single	5 (2.5)	4 (2.0)	3 (1.9)	1 (.7)	4 (2.6)	2 (1.4)
Other family situation	6 (3.0)	3 (1.5)	4 (2.6)	1 (.7)	4 (2.6)	1 (.7)
Mental illness ^a^, *n* (%)	41 (20.3)	43 (21.3)	32 (20.5)	31 (23.0)	27 (17.6)	35 (25.0)
Fear of birth counseling ^b^, *n* (%)	4 (2.0)	7 (3.5)	4 (2.6)	2 (1.5)	4 (2.6)	6 (4.3)
BMI at first antenatal visit,						
Mean (*SD*)	23.4 (3.4)	24.2 (4.1)	23.5 (3.4)	24.1 (3.5)	23.4 (3.5)	24.1 (3.7)
Median (IQR)	22.7 (20.9, 25.4)	23.1 (21.3, 26.4)	22.9 (21.1, 25.5)	23.1 (21.5, 25.9)	22.7 (20.9, 25.5)	23.3 (21.4, 25.7)
Birth preparation course ^c^, *n* (%)	155 (76.7)	161 (79.7)	127 (81.4)	115 (85.2)	125 (81.7)	117 (83.6)
Support person present, *n* (%)						
Partner	195 (96.5)	195 (96.5)	151 (96.8)	133 (98.5)	148 (96.7)	137 (97.9)
Doula	4 (2.0)	3 (1.5)	1 (.6)	1 (.7)	2 (1.3)	2 (1.4)
Other	14 (6.9)	21 (10.4)	9 (5.8)	5 (3.7)	9 (5.9)	9 (6.4)
Nobody	1 (.5)	0 (0)	1 (.6)	0 (0)	1 (.7)	0 (0)
Labor and birth outcomes						
Mode of birth, *n* (%)						
Vaginal spontaneous	169 (83.7)	171 (84.7)	129 (82.7)	114 (84.4)	124 (81.0)	119 (85.0)
Vaginal instrumental	19 (9.4)	15 (7.4)	17 (10.9)	11 (8.1)	17 (11.1)	8 (5.7)
Caesarean birth	14 (6.9)	16 (7.9)	10 (6.4)	10 (7.4)	12 (7.8)	13 (9.3)
Oxytocin augmentation, *n* (%)	98 (48.5)	117 (57.9)	77 (49.4)	80 (59.3)	76 (49.7)	83 (59.3)
Epidural analgesia, *n* (%)	109 (54.0)	132 (65.3) *	90 (57.7)	94 (69.6) *	88 (57.5)	95 (67.9)
Bath during labor, *n* (%)	119 (58.9)	59 (29.2) *	93 (59.6)	40 (29.6) *	88 (57.5)	40 (28.6) *
Length of labor ^d^ (hours)						
Mean (*SD*)	9 (5.1)	9.5 (5.0)	8.9 (5.0)	9.7 (4.9)	9 (5.1)	9.6 (4.8)
Median (IQR)	8.4 (4.6, 12.8)	9.1 (5.8, 12.4)	8.5 (4.6, 13)	9.2 (6.5, 12.9)	8.5 (4.6, 12.9)	9.6 (5.9, 12.4)
PP blood loss < 1,000 mL, *n* (%)	177 (87.6)	183 (90.6)	139 (89.1)	121 (89.6)	136 (88.9)	124 (88.6)
No severe vaginal laceration ^e^, *n* (%)	174/188 (92.6)	174/186 (93.5)	137/146 (93.8)	116/125 (92.8)	131/141 (92.9)	116/127 (91.3)
Hospital stay ^f^ (hours)						
Mean (*SD*)	59.7 (23.3)	59.1 (21.8)	59.2 (23.2)	57.5 (20.7)	60.9 (23.7)	59.1 (23.1)
Median (IQR)	55.3 (45.0, 70.5)	56.1 (45.6, 69.5)	54.9 (43.1, 70.6)	54.6 (43.6, 67.4)	56.4 (47.1, 71.3)	56.4 (43.4, 69.3)
NICU admission, n (%)	7 (3.5)	15 (7.4)	6 (3.8)	8 (5.9)	5 (3.3)	10 (7.1)
Apgar score < 7 at 5 min, *n* (%)	0 (0)	5 (2.5)	0 (0)	2 (1.5)	0 (0)	4 (2.9)

*Note.* BMI = body mass index; IQR = interquartile range; NICU = neonatal intensive care unit; PP = postpartum; *SD* = standard deviation.

^a^ Documented treatment due to mental illness. ^b^ Counseling at a specialized fear of birth clinic. ^c^ Attending pregnancy yoga, information at the hospital, or other childbirth preparation courses. ^d^ From randomization to time of birth. ^e^ No second degree vaginal injury in need of obstetric surgery, third or fourth degree anal sphincter injury. Not applicable if caesarean birth. ^f^ From time of birth to maternal hospital discharge.

* Statistically significant difference between groups.

### Self-Reported Childbirth Experience

The women’s overall childbirth experience on a VAS-OCE 2 hr after birth was similar in both randomized groups, as previously presented (Goldkuhl, Gyllensten et al., 2022). Women in the new room reported a more positive childbirth experience by higher scores at 3 months (*r* = .18, *p* = .002) and 12 months (*r* = .13*, p* = .021) after birth, compared to women in the regular room (Table 2). The differences in the two FOBS scores (*fear during labor* and *fear of a potential future birth*) between the groups were not statistically significant on either of the follow-up questionnaires (*p* > .05).

**Table 2.** Overall Childbirth Experience (VAS-OCE) and Fear of Birth (FOBS) 2 hr, 3 Months, and 12 Months After Birth.

Variables	2-Hr Follow-Up				3-Month Follow-Up					12-Month Follow-Up		
	New Room (*n* = 202)	Regular Room (*n* = 202)	Effect Size ^a^	*p*	New Room (*n* = 156)	Regular Room (*n* = 135)	Effect Size ^a^	*p*	New Room (*n* = 153)	Regular Room (*n* = 140)	Effect Size ^a^	*p*
Overall childbirth experience ^b^												
Mean (*SD*)	8.2 (2.0)	8.2 (1.8)			8.5 (2.0)	7.8 (2.3)			8.0 (2.4)	7.4 (2.6)		
Median (IQR)	9.0 (7.0, 10.0)	8.0 (7.0, 10.0)	.04	.384	9.2 (7.7, 10.0)	8.3 (6.7, 10.0)	.18	.002	8.8 (7.5, 10.0)	8.1 (6.3, 9.7)	.13	.021
95% CI for mean	[7.9, 8.5]	[7.9, 8.4]			[8.2, 8.8]	[7.5, 8.2]			[7.6, 8.4]	[7.0, 7.9]		
Fear during labor ^c^												
Mean (*SD*)	32.6 (25.2)	34.7 (24.1)			22.6 (26.2)	28.7 (29.4)			25.0 (27.5)	30.0 (29.4)		
Median (IQR)	26.8 (12.9, 46.6)	28.8 (14.0, 51.0)	.06	.233	13.3 (0.0, 33.9)	16.5 (4.0, 54.0)	.10	.075	15 (3, 38)	21.8 (4, 48)	.09	.146
95% CI for mean	29.1, 36.1	31.3, 38.0			18.4, 26.7	23.7, 33.7			20.6, 29.4	25.1, 34.9		
Cutoff > 60, *n* (%)	34 (16.8)	32 (15.8)		.788	21 (13.5)	30 (22.2)		.050	25 (16.3)	29 (20.7)		.335
Fear of future birth ^c^												
Mean (*SD*)	17.2 (12.4)	18.3 (11.9)			28.7 (28.8)	32.7 (31.8)			31.8 (30.6)	32.8 (34.2)		
Median (IQR)	14.4 (7.4, 24.3)	15.4 (8.0, 26.5)	.06	.233	17.8 (4.1, 46.5)	21 (6.0, 58.5)	.05	.361	23 (4, 56)	17.8 (2, 62)	.01	.866
95% CI for mean	15.5, 18.9	16.6, 19.9			24.2, 33.3	27.2, 38.1			29.9, 36.7	27.1, 38.5		
Cutoff > 60, *n* (%)	0 (0)	0 (0)		NA	28 (17.9)	32 (23.7)		.226	34 (22.2)	36 (25.7)		.484

*Note. p* values were calculated with Mann Whitney *U* test for continuous variables. For categorical variables, *p* values were calculated with χ^2^ test. All analyses were performed on the intention to treat population. CI = confidence interval; IQR = interquartile range; NA = not applicable; *SD* = standard deviation.

^a^ Effect size *r:* >.1 = *small effect*, >.3 = medium effect, >.5 = large effect. ^b^ Measured with a VAS cale of Overall Childbirth Experience 1–10. ^c^ Measured with the FOBS.

Women in the new room scored higher within the CEQ2 domain *own capacity* (*r* = .13, *p* = .028) and in the total CEQ2 score (*r* = .12, *p* = .039) 3 months after birth (Table 3). No significant differences between the groups was observed in the other three domains (*perceived safety*, *professional support*, and *participation*) at this time point (*p* > .05). There were no significant differences in any of the domains or in the total CEQ2 score between the groups 12 months after birth. Cronbach’s α coefficients for each of the four CEQ2 domains indicated internal consistency for the 3-month follow-up (new room vs. regular room); *own capacity*: α = .81 versus .80, *perceived safety*: α = .85 versus .86, *professional support*: α = .75 versus .85, and *participation*: α = .71 versus .74. Internal consistency was also seen for the 12-month follow-up; *own capacity*: α = .84 versus .84, *perceived safety*: α = .88 versus .89, *professional support*: α = .85 versus .82, and *participation*: α = .73 versus .85.

**Table 3.** Participants’ Childbirth Experience Questionnaire (CEQ2) Scores 3 And 12 Months After Birth.

Variables	3-Month Follow-Up				12-Month Follow-Up			
	New Room (*n* = 156)	Regular Room (*n* = 135)	Effect Size ^a^	*p*	New Room (*n* = 153)	Regular Room (*n* = 140)	Effect Size ^a^	*p*
Own capacity								
Mean (*SD*)	2.9 (.6)	2.7 (.6)			2.8 (.6)	2.7 (.6)		
Median (IQR)	3 (2.5, 3.4)	2.9 (2.3, 3.1)	.13	.028	2.9 (2.5, 3.3)	2.8 (2.1, 3.2)	.10	.081
95% CI for mean	[2.8, 3.0]	[2.6, 2.8]			[2.7, 2.9]	[2.6, 2.8]		
Perceived safety								
Mean (*SD*)	3.4 (.7)	3.3 (.7)			3.3 (.7)	3.2 (.8)		
Median (IQR)	3.7 (3.2, 3.8)	3.5 (3.0, 3.8)	.10	.094	3.7 (3.0, 3.8)	3.5 (2.7, 3.8)	.08	.154
95% CI for mean	[3.3, 3.5]	[3.2, 3.4]			[3.2, 3.4]	[3.1, 3.3]		
Professional support								
Mean (*SD*)	3.6 (.5)	3.6 (.6)			3.5 (.6)	3.5 (.6)		
Median (IQR)	3.8 (3.4, 4.0)	3.8 (3.4, 4.0)	.03	.568	3.8 (3.4, 4.0)	3.6 (3.2, 4.0)	.10	.093
95% CI for mean	[3.6, 3.7]	[3.5, 3.7]			[3.4, 3.6]	[3.4, 3.6]		
Participation								
Mean (*SD*)	3.6 (.6)	3.5 (.6)			3.5 (.7)	3.4 (.7)		
Median (IQR)	4 (3.3, 4.0)	3.7 (3.3, 4.0)	.11	.070	3.7 (3.3, 4.0)	3.7 (3.0, 4.0)	.03	.650
95% CI for mean	[3.5, 3.7]	[3.4, 3.6]			[3.4, 3.6]	[3.3, 3.6]		
Total CEQ2 score								
Mean (*SD*)	3.4 (.5)	3.3 (.5)			3.3 (.6)	3.2 (.6)		
Median (IQR)	3.5 (3.3, 3.7)	3.4 (3.0, 3.7)	.12	.039	3.4 (3.1, 3.7)	3.4 (2.9, 3.6)	.10	.091
95% CI for mean	[3.3, 3.5]	[3.2, 3.4]			[3.2, 3.4]	[3.1, 3.3]		

*Note. p* values were calculated with Mann Whitney *U* test in each of the four CEQ2 domains as well as the Total CEQ2 score. All analyses were performed on the intention to treat population. CEQ2 = Childbirth Experience Questionnaire Version 2; CI = confidence interval; IQR = interquartile range; *SD* = standard deviation.

^a^ Effect size *r:* >.1 = small effect, >.3 = medium effect, and >.5 = large effect.

### Childbirth Experience Measured Over Time

**Figure 5.**
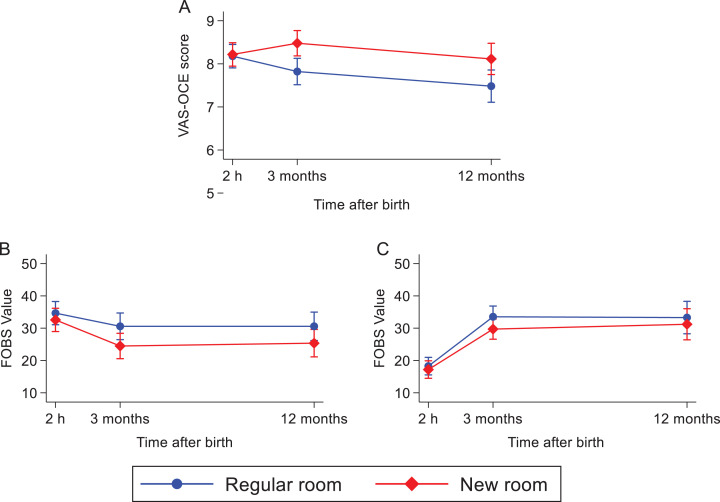
Childbirth experience 2 hr, 3 months, and 12 months after birth in the two randomized groups measured with a linear mixed-effects model. (A) Visual Analogue Scale of Overall Childbirth Experience (VAS-OCE) 1–10, (B) fear during past childbirth on a Fear of Birth Scale (FOBS) 0–100, and (C) fear of giving birth again on a FOBS 0–100.

**Figure 6.**
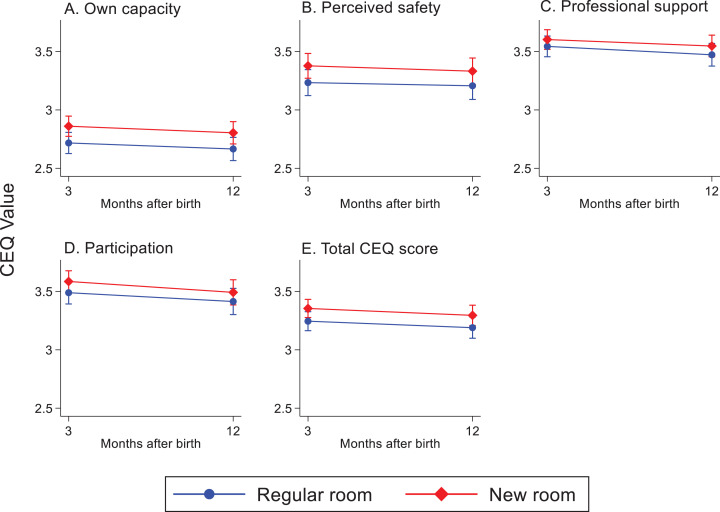
Childbirth Experience Questionnaire (CEQ2) values (1–4) within the four domains (*own capacity*, *perceived safety*, *professional support*, and *participation*) and in the total CEQ score. Reported over time in the two randomized groups. Measured with a linear mixed-effects model.

The childbirth experience scored on a VAS-OCE decreased at 3 months (*B* = −0.36, *p* = .005) and 12 months (*B* = −0.70, *p* < .001) after birth among women in both randomized groups (Supplementary Information 3). Women allocated to the new room scored higher in overall childbirth experience over time than women in the regular room (3 months: *B* = .62, *p* < .001 and 12 months: *B* = .59, *p* = .012; Figure 5). There were no differences between the groups regarding fear during childbirth over time, but the total cohort had a reduced experience of fear when thinking back 3 months after their recent birth (*B* = −4.08, *p* = .035) and these levels of fear remained after 12 months. In contrast, the reported fear of a potential future birth increased in the total cohort after 3 months (*B* = 15.28, *p* < .001) and remained after 12 months. This increase in reported fear did not differ between the randomized groups (Figure 5). For the CEQ2 scores reported 3 and 12 months after birth, the total cohort reported decreased scores over time within the domains *professional support* (*B* = −0.07, *p* = .019), *participation* (*B* = −0.08, *p* = .030), and for the total CEQ2 score (*B* = −0.06, *p* = .005). The change over time did not differ between the randomized groups in the total CEQ2 score or in any of the subdomains (Supplementary Information 3; Figure 6).

The significant effect of the new room in the CEQ2 subdomain *own capacity* remained after our subsequent analysis controlling for the use of epidural analgesia (*B* = .13, *p* = .047), but not for oxytocin augmentation (*B* = .12, *p* = .061; Supplementary Information 4). These adjusted analyses also showed slightly reduced *B*-coefficients considering the effect of the room on the VAS-OCE among all responders randomized to the new room. The difference regarding time and the interaction between time and the randomized group did not change after the subsequent analyses in any of the outcome variables (VAS-OCE, FOBS, and CEQ2).

## Discussion

This randomized trial shows that a hospital birthing room designed with person-centered considerations offering women possibilities to change features and functions in the room has a positive impact on the self-reported childbirth experience 3 and 12 months after birth. Women randomized to the new room had a more positive overall childbirth experience as reported on a VAS-OCE, 3 and 12 months after birth, compared with women randomized to a regular room. Study participants in the new room also had significantly higher scores in the CEQ2 domain *own capacity* and total CEQ2 score 3 months after birth, which additionally indicates more positive overall childbirth experiences. The statistically significant differences between the groups regarding the CEQ2 variables did not remain in the 12-month follow-up.

***This randomized trial shows that a hospital birthing room designed with person-centered considerations offering women possibilities to change features and functions in the room has a positive impact on the self-reported childbirth experience 3 and 12 months after birth***.

To our knowledge, this is the first quantitative evaluation of the effect of a specially designed hospital birthing room on women’s childbirth experience over several time points during the first year after birth. The study findings emphasize that the physical design of the room is one essential contributing factor for subsequent childbirth satisfaction, which supports our hypothesis. This is also in accordance with previous findings about the experiential value of a conscientiously designed birthing room (Nielsen & Overgaard, 2020; Skogström et al., 2022). An explanation of these results may be that the new room was designed based on the hypothesized needs of birthing women (Nilsson et al., 2020) and not primarily on streamlining institutional needs. Neither was the room designed based on a technocratic view of childbirth as being a critical event but on a more holistic view and a striving to optimize the release of oxytocin that is known to physiologically reduce stress and affect emotional well-being (Uvnas-Moberg et al., 2019). These stress-reducing elements within the new room, thereby, seemed to impact the women’s childbirth experience 3 and 12 months after birth. It has previously been described how a nonclinical-like, familiarized and personalized birth environment may influence women’s practices and perceptions during labor. For instance, an environment that facilitates upright birth position, physical comfort, and positive distraction, such as the music and nature films displayed in the new room, is beneficial for coping with stress and labor pain (Andrade & Devlin, 2015).

Women randomized to the new room reported higher scores within the CEQ2 domain *own capacity* after 3 months, which indicates that they experienced a greater sense of self-efficacy and control during birth than women in the regular room. This may have been due to women in the new room being provided with more opportunities to modify physical aspects of the birth environment. Women may experience this as being allowed to prepare the environment for giving birth or, when expressing it from a biological perspective, to prepare the nest for the baby via expression of archaic patterns of behavior. This procedure is essential for birthing women and most certainly, in a subtle way, increases the sense of familiarity with the room, thereby enhancing oxytocin release and the experience of positive emotions (Uvnas-Moberg et al., 2019). As shown in previous research, a sense of personal influence over the surroundings and involvement in labor and birth processes are essential for a positive childbirth experience (Townsend et al., 2020).

***Women randomized to the new room reported higher scores within the CEQ2 domain *own capacity* after 3 months, which indicates that they experienced a greater sense of self-efficacy and control during birth***.

Our findings showed that there were no clear differences in women’s reported fear of birth between the groups, although slightly more women in the regular room had FOBS scores above the cutoff point of 60, indicating fear when reflecting about the childbirth experience 3 months later. Neither were there any significant differences in the CEQ2 subdomains *perceived safety* and *participation* between the groups. It is well established from previous research that social support during labor is a key component in experiencing childbirth satisfaction, which undeniably cannot be compensated by the design of the room (Olza et al., 2020). Our results show that the new room had no effect on the CEQ2 subdomain *professional support*, which indicates that the provided care did not differ between the two groups. Previous research has provided knowledge of how the physical design has the potential of enabling healthcare providers to support women during physiological birth (Andrén et al., 2021). In one of our earlier studies, we have described how a room that conveys a sense of comfort, familiarity, and integrity has the potential to symbolize tenderness and care for birthing women in an otherwise unfamiliar hospital context (Goldkuhl, Dellenborg et al., 2022). However, this did not have any effect on the reported CEQ2 scores within the *professional support* domain in the present study.

The measures of childbirth experience on a VAS-OCE in the two randomized groups differed 3 and 12 months after birth, but not immediately after birth as we originally hypothesized. Women in the regular room reported a more negative birth experience after 3 and 12 months, while the women in the new room reported a more positive experience after 3 months than in their immediate response 2 hr after birth. It has previously been recognized that the childbirth experience generally becomes more negative over time, though women tend to forget the intensity of the labor pain (Waldenström & Schytt, 2009). One explanation of this may be that a response made 2 hr after birth does not reflect the overall perception of the experience. Women tend to base their long-term assessments more on interventions and birth outcomes than the immediate relief and joy of having completed the birth of a healthy baby and the satisfaction with the provided professional support (Turkmen et al., 2018). In addition, the amnesic effect of oxytocin, by which negative experiences are attenuated, develops gradually and is not necessarily present directly after birth (Olza et al., 2020; Uvnas-Moberg et al., 2019).

***Women in the regular room reported a more negative birth experience after 3 and 12 months, while the women in the new room reported a more positive experience after 3 months than in their immediate response 2 hr after birth***.

Women in both randomized groups reported lower levels of fear during birth when thinking back 3 and 12 months than when responding immediately after birth. Conversely, their fear of giving birth again increased over time. This is consistent with previous research that used the FOBS to retrospectively measure fear of birth (Rondung et al., 2018) and of a potential future birth (Hildingsson, 2021). It is well known that a negative or traumatic childbirth experience is correlated with an increased fear of giving birth again (Dencker et al., 2019). Since women’s overall childbirth experience becomes more negative over time (Waldenström, 2004), this may be an explanation of the increased fear of a potential future birth. However, our results indicate that the physical design of the birthing room does not seem to affect the experience of childbirth fear.

### Methodological Considerations

The advantages of our study include its randomized design and the high response rates of the follow-up questionnaires. Another strength is that the participants were asked to report their childbirth experiences at three different time points during their first year after birth. This made it possible to study how the experience of birth changes over time. Unfortunately, this study had a limited sample size, which was not sufficient to detect an effect in the 12-month follow-up. The sample size estimation was based on the primary outcome of the Room4Birth RCT (Goldkuhl, Gyllensten et al., 2022), which had an early termination due to the Covid-19 pandemic. Since the current study presents a secondary outcome, a power calculation for the childbirth experiences up to 1 year after birth was not performed. Another study limitation was that we had no valid translations for the follow-up questionnaires 2 and 3 in Arabic and Somali. However, two of these three participants did answer *Follow-Up 3* in Swedish.

To explore the multidimensional phenomenon of childbirth experience, we chose to include three different measurements (VAS-OCE, FOBS, and CEQ2). Nevertheless, it is challenging to measure experiences quantitatively, since aspects such as context, social support, interpersonal relationships, and physical and emotional senses need to be taken into consideration (Hall et al., 2018). A study limitation is that the modification of FOBS that was used in this study is not validated. VAS-OCE may also be a one-dimensional measurement that does not provide sufficient information to comprehensively understand all aspects of the phenomenon of a childbirth experience. However, both measurements have been recurrently used in previous studies (Falk et al., 2019; Hildingsson, 2021; Nilver et al., 2021; Rondung et al., 2018), and this quantitative exploration may provide knowledge that can be used as a complement to qualitative research.

The place and space for birth is not just created through the physical design but also through those who inhabit and interact within the room (Lefebvre, 1991). Although the intention was that the study participants, regardless of allocated room, would be provided with the same level of care, it is possible that care providers were also affected by the design of the room. It is known that professional support is a main contributing factor for experiencing satisfaction with childbirth (Bohren et al., 2017), and the open-labeled design of the current trial might, therefore, be a study disadvantage. It might be that the calming environment in the new room supported care providers in their care practices (Hammond et al., 2014). On the other hand, this trial was conducted in a large hospital-based labor ward with many employees of several different professions, all of whom were not comfortable with working in the alternative design of the new room. They were all given the opportunity to get familiar with the room and use it for a few weeks before data collection for the main trial started. However, this acclimatization period might have been too limited to allow them to be completely familiar with the room design.

## Conclusion

The findings of this study show the benefit of acknowledging the physical design of hospital-based birthing rooms. Nulliparous women randomized to a birthing room designed with person-centered considerations aimed at supporting the physiology of birth reported more positive overall childbirth experiences 3 and 12 months after birth than women randomized to a regular birthing room. When designing interventions and environments in hospital settings, it is essential to understand the psychological and existential dimensions of childbirth, since the dynamics and changes in labor and birth experiences influence outcomes. It is also essential to understand how remnants of archaic nest-building behaviors and experiences are still present in birthing women of today. The findings of the study confirm that the physical design matters to women in their assessment of childbirth experience, but we were unable to show the actual effect of the new room on the variables explored. Therefore, additional research is needed that qualitatively explores the impact of the birth environment design on women’s long-term childbirth experiences.

## Implications for Practice

- From a mental health perspective, it is essential to understand the importance of emotional well-being in the transition to parenthood.
- A birthing room designed with person-centered considerations, where women and companions can adapt features and functions in the room in accordance with personal needs and requirements, positively impacts women’s birth experiences 3 and 12 months after birth.
- When striving to optimize care during labor and birth, both the physical and the psychosocial environment need to be improved.
- To value emotions, birth physiology and person-centered perspectives in labor ward design may be one strategy to help reverse the rising trend of medical interventions in labor that is observed in many hospital settings.

## Supplemental Material

Supplemental Material, sj-pdf-1-her-10.1177_19375867221124232 - Impact of Birthing Room Design on Maternal Childbirth Experience: Results From the Room4Birth Randomized Trial Click here for additional data file. Supplemental Material, sj-pdf-1-her-10.1177_19375867221124232 for Impact of Birthing Room Design on Maternal Childbirth Experience: Results From the Room4Birth Randomized Trial by Lisa Goldkuhl, Hanna Gyllensten, Cecily Begley, Christina Nilsson, Helle Wijk, Göran Lindahl, Kerstin Uvnäs-Moberg and Marie Berg in HERD: Health Environments Research & Design Journal
