# Supplementary material for: Impact of Birthing Room Design on Maternal Childbirth Experience: Results From the Room4Birth Randomized Trial
Source: HERD. 2022 Oct 14;16(1):200–18. doi: 10.1177/19375867221124232 (PMC9755691; doi:10.1177/19375867221124232)
Supplement: Supplemental Material, sj-pdf-1-her-10.1177_19375867221124232 - Impact of Birthing Room Design on Maternal Childbirth Experience: Results From the Room4Birth Randomized Trial [file sj-pdf-1-her-10.1177_19375867221124232.pdf]

Supplementary information 1-5

Impact of Birthing Room Design on Maternal Childbirth Experience: Results from the Room4Birth Randomised Trial

Lisa Goldkuhl, MSc, RN, RM[1,2]*, Hanna Gyllensten, PhD, MScPharm[1,3], Cecily Begley, PhD, RM[4], Christina Nilsson, PhD, RN, RM[5], Helle Wijk, PhD, RN[1,6,7,8], Göran Lindahl, PhD, M.Arch[7,8], Kerstin Uvnäs-Moberg, PhD, MD[9], Marie Berg, PhD, RN, RM[1,2, 10]

[1] Institute of Health and Care Sciences, Sahlgrenska Academy, University of Gothenburg, Gothenburg, Sweden
[2] Department of Obstetrics and Gynaecology, Sahlgrenska University Hospital, Gothenburg, Region Västra Götaland, Sweden
[3] University of Gothenburg Centre for Person-Centred Care (GPCC), Sahlgrenska Academy, University of Gothenburg, Gothenburg, Sweden
[4] School of Nursing and Midwifery, Trinity College Dublin, The University of Dublin, Dublin, Ireland
[5] Munkebäck Antenatal Clinic, Region Västra Götaland, Gothenburg, Sweden
[6] Department of Quality Assurance and Patient Safety, Sahlgrenska University Hospital, Region Västra Götaland, Gothenburg, Sweden
[7] Centre for Healthcare Architecture, CVA, Chalmers University of Technology, Gothenburg, Sweden
[8] Department of Architecture and Civil Engineering, Building Design, Chalmers University of Technology, Gothenburg, Sweden
[9] University of Agriculture (SLU), Uppsala University, Uppsala, Sweden
[10] Faculty of Medicine and Community Health, Evangelical University of Africa, Bukavu, D.R Congo

Supplementary information

1. Digital questionnaire answered two hours after birth (*Follow-up 1*) Page 2
2. Digital questionnaire answered 3 and 12 months after birth (*Follow-up 2 and 3*) Page 7
3. Table 1. Linear mixed effects model measuring childbirth experience over time. Total ITT study sample Page 12
4. Table 2. Results from the Linear mixed effects model controlling for oxytocin augmentation and epidural analgesia on the dependent variable: Overall childbirth experience on a VAS 1-10. Page 14

   Table 3. Results from the Linear mixed effects model controlling for oxytocin augmentation and epidural analgesia on the dependent variable: CEQ2 Own capacity. Page 14

   Table 4. Results from the Linear mixed effects model controlling for oxytocin augmentation and epidural analgesia on the dependent variable: Total mean CEQ2. Page 14
5. CONSORT checklist Page 15

Supplementary information 1.

Digital questionnaire answered by the participants two hours after birth

**1)**	**Nummer**	**ID**
		1000-6000

**2)**	**Enval**	**Randomised room answered by the care provider**
		1) New room (Room 0)
		2) Regular room (Room 3-9)

**3)**	**Nummer**	**Age**
		18-60

**4)**	**Enval**	**Education**
		1) Compulsory / elementary school (year 1-9 or equivalent)
		2) High school (year 10-12 or equaivalent)
		3) University or College
	Information:	*(ange högsta) (highest reached level)*

**5)**	**Enval**	**Which is your current family situation?**
		1) Cohabiting with the other parent
		2) Single
		3) other family situation

**6)**	**Enval**	**In which country were you born?**
		1) Sweden
		2) Other country

**7)**	**Text**	**In which country were you born?**
	Villkor:	*6=2*

8)	Nummer	**What year did you arrive to Sweden?**
	Villkor:	*6=2*

9)	Flerval	
	Information:	**Have you participated in a childbirth preparation course or taken part in information about labour?**<br>1) Childbirth preparation course (for example ”Magplasket”, ”Föda Utan Rädsla”, Pregnant yoga or in any other childbirth preparation course)<br>2) Information at the hospital Östra Sjukhuset<br>3) Have not participated in any of the above<br>*(you can choose several alternatives)*

10)	Flerval	
	Information:	**Who was with you during labour apart from the health professionals?**<br>1) Partner<br>2) Doula<br>3) Other person<br>4) Nobody else was with me<br>*(you can choose several alternatives)*

11)	Betyg	**How do you rate your overall childbirth experience?**<br>1) 1, Very bad<br>2) 2<br>3) 3<br>4) 4<br>5) 5<br>6) 6<br>7) 7<br>8) 8<br>9) 9<br>10) 10, Very Good

12)	Nummer (liggande streck)	**How do you rate worry and fear when you THINK ABOUT YOUR COMPLETED LABOUR AND BIRTH? Please mark with an X on the lines below**<br>Calm ———————— Worried

**13)**	**Nummer (liggande streck)**	**How do you rate worry and fear when you THINK ABOUT YOUR COMPLETED LABOUR AND BIRTH? Please mark with an X on the lines below**<br>No fear ______________________ Strong fear

**14)**	**Nummer (liggande streck)**	**How do you feel right now ABOUT GIVING BIRTH AGAIN? Please mark with an X on the lines below**<br>Calm ______________________ Worried

**15)**	**Nummer (liggande streck)**	**How do you feel right now ABOUT GIVING BIRTH AGAIN? Please mark with an X on the lines below**<br>No fear ______________________ Strong fear

**16)**	**Enval**	
		**To what extent did the room design contribute to your sense of safety?**<br>1) To a very high degree<br>2) To a high degree<br>3) To a low degree<br>4) Not at all<br>*SAFETY*

**17)**	**Enval**	**How important is this for you**<br>1) Very important<br>2) Important<br>3) Slightly important<br>4) Not important

**18)**	**Enval**	
	Rubrik:	**To what extent did the room design contribute to your sense of control?**<br>1) To a very high degree<br>2) To a high degree<br>3) To a low degree<br>4) Not at all<br>*CONTROL*

19) **Enval** **How important is this for you**

1) Very important
2) Important
3) Slightly important
4) Not important

20) **Enval**

To what extent did the room design contribute to your sense of integrity?

1) To a very high degree

2) To a high degree

3) To a low degree

4) Not at all

INTEGRITY

21) **Enval** **How important is this for you**

1) Very important
2) Important
3) Slightly important
4) Not important

22) **Enval**

To what extent did the room design adapt to your needs and requests?

1) To a very high degree

2) To a high degree

3) To a low degree

4) Not at all

ADAPTABILITY

23) **Enval** **How important is this for you**

1) Very important
2) Important
3) Slightly important
4) Not important

**24)**	**Enval**	
		**Were the functions in the birthing room meaningful for your birth experience?**<br><br>1) To a very high degree<br><br>2) To a high degree<br><br>3) To a low degree<br><br>4) Not at all<br><br>*MEANINGFULNESS, THE FUNCTIONS OF THE BIRTH ROOM*

**25)**	**Enval**	How important is this for you<br><br>1) Very important<br>2) Important<br>3) Slightly important<br>4) Not important

Supplementary information 2.

Digital questionnaire answered 3 and 12 months after birth (*Follow-up 2 and 3*)

CHILDBIRTH EXPERIENCE

1. How was your overall childbirth experience?

(Indicate your opinion by marking on the line between the two end-points)

Very bad ●————————————————● Very good

2. Labour and birth went as I had expected.

(Please tick the box below the response choice that best corresponds to your opinion)

Totally agree	Mostly agree	Mostly disagree	Totally disagree
☐	☐	☐	☐

3 . I felt strong during labour and birth.

Totally agree	Mostly agree	Mostly disagree	Totally disagree
☐	☐	☐	☐

4. I felt scared during labour and birth.

Totally agree	Mostly agree	Mostly disagree	Totally disagree
☐	☐	☐	☐

5. I felt capable during labour and birth.

Totally agree	Mostly agree	Mostly disagree	Totally disagree
☐	☐	☐	☐

6. I was tired during labour and birth.

Totally agree	Mostly agree	Mostly disagree	Totally disagree
☐	☐	☐	☐

7. I felt happy during labour and birth.

Totally agree	Mostly agree	Mostly disagree	Totally disagree
☐	☐	☐	☐

8. I felt that I handled the situation well.

Totally agree	Mostly agree	Mostly disagree	Totally disagree
☐	☐	☐	☐

9. I would have preferred another form of pain relief.

Totally agree	Mostly agree	Mostly disagree	Totally disagree
☐	☐	☐	☐

10. I wish the staff had listened to me more during labour and birth.

Totally agree	Mostly agree	Mostly disagree	Totally disagree
☐	☐	☐	☐

11. I could get up and move around as much as I wanted.

Totally agree	Mostly agree	Mostly disagree	Totally disagree
☐	☐	☐	☐

12. I could give birth in the way I wanted.

Totally agree	Mostly agree	Mostly disagree	Totally disagree
☐	☐	☐	☐

13. I took part in decisions regarding my care and treatment as much as I wanted.

Totally agree	Mostly agree	Mostly disagree	Totally disagree
☐	☐	☐	☐

14. Both my partner and I were treated with warmth and respect.

Totally agree	Mostly agree	Mostly disagree	Totally disagree
◻	◻	◻	◻

15. I received the information I needed during labour and birth.

Totally agree	Mostly agree	Mostly disagree	Totally disagree
◻	◻	◻	◻

16. I would have preferred the midwife to be more present during labour and birth.

Totally agree	Mostly agree	Mostly disagree	Totally disagree
◻	◻	◻	◻

17. I would have preferred more encouragement from the midwife.

Totally agree	Mostly agree	Mostly disagree	Totally disagree
◻	◻	◻	◻

18. The midwife conveyed an atmosphere of calm.

Totally agree	Mostly agree	Mostly disagree	Totally disagree
◻	◻	◻	◻

19. The midwife helped me to find my inner strength.

Totally agree	Mostly agree	Mostly disagree	Totally disagree
◻	◻	◻	◻

20. My impression of the team’s medical skills made me feel secure.

Totally agree	Mostly agree	Mostly disagree	Totally disagree
◻	◻	◻	◻

22. I have many positive memories from childbirth.

Totally agree	Mostly agree	Mostly disagree	Totally disagree
☐	☐	☐	☐

22. I have many negative memories from childbirth.

Totally agree	Mostly agree	Mostly disagree	Totally disagree
☐	☐	☐	☐

23. Some of my memories from childbirth make me feel depressed.

Totally agree	Mostly agree	Mostly disagree	Totally disagree
☐	☐	☐	☐

24. As a whole, how painful did you feel childbirth was?

(Indicate your opinion by marking on the line between the two end-points)

No pain —— Worst imaginable pain

25. As a whole, how much control did you feel you had during childbirth?

No control —— Complete control

26. As a whole, how secure did you feel during childbirth?

Not at all secure —— Completely secure

FEAR OF BIRTH

28. How do you rate worry and fear when you think about your completed labour and birth?

(Indicate your opinion by marking on the line between the two end-points)

Calm ●——————————————● Worried

29. How do you rate worry and fear when you think about your completed labour and birth?

No fear ●——————————————● Strong fear

30. How do you rate worry and fear when you think about giving birth again?

Calm ●——————————————● Worried

31. How do you rate worry and fear when you think about giving birth again?

No fear ●——————————————● Strong fear

Supplementary information 3

Table 1. Linear mixed effects model measuring childbirth experience over time. Total ITT study sample

Variables	Beta (SE)	*P*	95% LCI	95% UCI	Random effect, Beta (95% LCI; UCI)
Overall childbirth experience[a]					
Time					0.01 (0.01; 0.02)
3 months[b]	-0.36 (0.13)	**0.005**	-0.61	-0.11	
12 months[b]	-0.70 (0.17)	**<0.001**	-1.03	-0.36	
New room[c]	0.04 (0.20)	0.840	-0.34	0.42	
3 months x New room[d]	0.62 (0.18)	**<0.001**	0.27	0.96	
12 months x New room[d]	0.59 (0.23)	**0.012**	0.13	1.05	
intercept	8.18 (0.14)	<0.001	7.91	8.45	
Fear during past childbirth[e]					
Time					0.74 (0.29; 1.89)
3 months[b]	-4.08 (1.94)	**0.035**	-7.88	-0.28	
12 months[b]	-4.08 (2.09)	0.051	-8.17	0.02	
New room[c]	-2.10 (2.60)	0.419	-7.19	2.99	
3 months x New room[d]	-3.99 (2.67)	0.134	-9.22	1.23	
12 months x New room[d]	-3.12 (2.90)	0.282	-8.80	2.56	
intercept	34.66 (1.84)	<0.001	31.06	38.26	
Fear of future birth[e]					
Time					3.98 (3.04; 5.23)
3 months[b]	15.28 (1.77)	**<0.001**	11.81	18.75	
12 months[b]	15.03 (2.61)	**<0.001**	9.92	20.15	
New room[c]	-1.05 (1.95)	0.590	-4.89	2.78	
3 months x New room[d]	-2.76 (2.44)	0.259	-7.54	2.03	
12 months x New room[d]	-1.02 (3.62)	0.779	-8.11	6.08	
intercept	18.25 (1.38)	<0.001	15.54	20.96	
CEQ2 Own capacity					
Time					0.00 (0.00; 0.00)
12 months[f]	-0.05 (0.03)	0.060	-0.11	0.00	
New room[c]	0.14 (0.06)	**0.025**	0.02	0.27	
12 months x New room[g]	-0.00 (0.04)	0.898	-0.08	0.07	
intercept	2.72 (0.05)	<0.001	2.63	2.81	
CEQ2 Perceived safety					
Time					0.00 (0.00; 0.00)
12 months[f]	-0.03 (0.03)	0.393	-0.09	0.03	
New Room[c]	0.14 (0.08)	0.067	-0.01	0.30	
12 months x New room[g]	-0.02 (0.04)	0.668	-0.10	0.07	
intercept	3.23 (0.06)	<0.001	3.12	3.34	
CEQ2 Professional support					
Time					0.00 (0.00; 0.00)
12 months[f]	-0.07 (0.03)	**0.019**	-0.13	-0.01	
New room[c]	0.06 (0.06)	0.347	-0.06	0.18	
12 months x New room[g]	0.02 (0.04)	0.692	-0.06	0.10	
intercept	3.54 (0.05)	<0.001	3.46	3.63	
CEQ2 Participation					
Time					0.00 (0.00; 0.00)
12 months[f]	-0.08 (0.03)	**0.030**	-0.14	-0.01	
New room[c]	0.10 (0.07)	0.156	-0.04	0.23	

12 months x New room[g]	-0.02 (0.05)	0.701	-0.11	0.07	
intercept	3.49 (0.05)	<0.001	3.39	3.59	
Total CEQ2 score					
Time					0.00 (0.00; 0.00)
12 months[f]	-0.06 (0.02)	**0.005**	-0.09	-0.02	
New room[c]	0.11 (0.06)	0.057	-0.00	2.22	
12 months x New room[g]	-0.00 (0.03)	0.886	-0.06	0.05	
intercept	3.24 (0.04)	<0.001	3.16	3.33	

CEQ2=Childbirth Experience Questionnaire version 2, LCI=Lower Confidence Interval, ITT=Intention To Treat, UCI=Upper Confidence Interval

[a] Measured with Visual Analogue Scale (VAS 1-10)

[b] Reference = 2 hours after birth

[c] Reference = Regular room

[d] Reference = 2 hours and Regular room

[e] Measured with the Fear of Birth Scale (FOBS)

[f] Reference = 3 months after birth

[g] Reference = 3 months and Regular room

Supplementary information 4

Table 2. Results from the Linear mixed effects model controlling for oxytocin augmentation and epidural analgesia on the dependent variable: Overall childbirth experience on a VAS 1-10.

	Model 1[a]	Model 2[b]	Model 3[c]
Variables	Beta	Beta	Beta
New room[d]	0.04	-0.05	-0.02
3 months	-0.36	-0.35	-0.35
12 months	-0.70	-0.69	-0.69
3 months x New room[e]	0.62	0.62	0.62
12 months x New room[e]	0.59	0.59	0.59
Intercept	8.18	8.70	8.52

VAS=Visual Analogue Scale,

[a] Unadjusted

[b] Adjusted for Oxytocin augmentation

[c] Adjusted for Epidural analgesia

[d] Reference = Regular room

[e] Reference = 2 hours and Regular room

Table 3. Results from the Linear mixed effects model controlling for oxytocin augmentation and epidural analgesia on the dependent variable: CEQ2 Own capacity.

	Model 1[a]	Model 2[b]	Model 3[c]
Variables	Beta	Beta	Beta
New room[d]	0.14	0.12	0.13
12 months	-0.05	-0.05	-0.05
12 months x New room[e]	-0.005	-0.005	-0.005
Intercept	2.72	2.86	2.80

CEQ2=Childbirth Experience Questionnaire version 2

[a] Unadjusted

[b] Adjusted for Oxytocin augmentation

[c] Adjusted for Epidural analgesia

[d] Reference = Regular room

[e] Reference = 3 months and Regular room

Table 4. Results from the Linear mixed effects model controlling for oxytocin augmentation and epidural analgesia on the dependent variable: Total mean CEQ2.

	Model 1[a]	Model 2[b]	Model 3[c]
Variables	Beta	Beta	Beta
New room[d]	0.11	0.09	0.10
12 months	-0.06	-0.06	-0.06
12 months x New room[e]	-0.004	-0.004	-0.004
Intercept	3.24	3.37	3.28

CEQ2=Childbirth Experience Questionnaire version 2

[a] Unadjusted

[b] Adjusted for Oxytocin augmentation

[c] Adjusted for Epidural analgesia

[d] Reference = Regular room

[e] Reference = 3 months and Regular room

Supplementary information 5

CONSORT 2010 checklist of information to include when reporting a randomised trial*

Section/Topic	Item No	Checklist item	Reported on page No
**Title and abstract**			
	1a	Identification as a randomised trial in the title	Title page
	1b	Structured summary of trial design, methods, results, and conclusions (for specific guidance see CONSORT for abstracts)	Abstract
**Introduction**			
Background and objectives	2a	Scientific background and explanation of rationale	1-2
	2b	Specific objectives or hypotheses	2
**Methods**			
Trial design	3a	Description of trial design (such as parallel, factorial) including allocation ratio	3
	3b	Important changes to methods after trial commencement (such as eligibility criteria), with reasons	7
Participants	4a	Eligibility criteria for participants	4
	4b	Settings and locations where the data were collected	3
Interventions	5	The interventions for each group with sufficient details to allow replication, including how and when they were actually administered	3-4
Outcomes	6a	Completely defined pre-specified primary and secondary outcome measures, including how and when they were assessed	5-7

	6b	Any changes to trial outcomes after the trial commenced, with reasons	
Sample size	7a	How sample size was determined	See study protocol or Main outcome paper
	7b	When applicable, explanation of any interim analyses and stopping guidelines	See study protocol
Randomisation:			
Sequence generation	8a	Method used to generate the random allocation sequence	4-5
	8b	Type of randomisation; details of any restriction (such as blocking and block size)	p. 4 and Main outcome paper
Allocation concealment mechanism	9	Mechanism used to implement the random allocation sequence (such as sequentially numbered containers), describing any steps taken to conceal the sequence until interventions were assigned	4-5
Implementation	10	Who generated the random allocation sequence, who enrolled participants, and who assigned participants to interventions	4-5
Blinding	11a	If done, who was blinded after assignment to interventions (for example, participants, care providers, those assessing outcomes) and how	4-5
	11b	If relevant, description of the similarity of interventions	4
Statistical methods	12a	Statistical methods used to compare groups for primary and secondary outcomes	7-8
	12b	Methods for additional analyses, such as subgroup analyses and adjusted analyses	8
**Results**			
Participant flow (a diagram is	13a	For each group, the numbers of participants who were randomly assigned, received intended treatment, and were analysed for the primary outcome	8, figure 4

strongly recommended)	13b	For each group, losses and exclusions after randomisation, together with reasons	8, figure 4
Recruitment	14a	Dates defining the periods of recruitment and follow-up	3
	14b	Why the trial ended or was stopped	Methodological considerations and Main outcome paper
Baseline data	15	A table showing baseline demographic and clinical characteristics for each group	Table 1
Numbers analysed	16	For each group, number of participants (denominator) included in each analysis and whether the analysis was by original assigned groups	8, Table 2 and 3
Outcomes and estimation	17a	For each primary and secondary outcome, results for each group, and the estimated effect size and its precision (such as 95% confidence interval)	Table 2 and 3
	17b	For binary outcomes, presentation of both absolute and relative effect sizes is recommended	-
Ancillary analyses	18	Results of any other analyses performed, including subgroup analyses and adjusted analyses, distinguishing pre-specified from exploratory	p. 10-11. Supplementary information 4
Harms	19	All important harms or unintended effects in each group (for specific guidance see CONSORT for harms)	Main outcome paper
**Discussion**			
Limitations	20	Trial limitations, addressing sources of potential bias, imprecision, and, if relevant, multiplicity of analyses	14-15
Generalisability	21	Generalisability (external validity, applicability) of the trial findings	14-15
Interpretation	22	Interpretation consistent with results, balancing benefits and harms, and considering other relevant evidence	11-13
**Other information**			

Registration	23	Registration number and name of trial registry	3
Protocol	24	Where the full trial protocol can be accessed, if available	3
Funding	25	Sources of funding and other support (such as supply of drugs), role of funders	Title page

*We strongly recommend reading this statement in conjunction with the CONSORT 2010 Explanation and Elaboration for important clarifications on all the items. If relevant, we also recommend reading CONSORT extensions for cluster randomised trials, non-inferiority and equivalence trials, non-pharmacological treatments, herbal interventions, and pragmatic trials. Additional extensions are forthcoming: for those and for up to date references relevant to this checklist, see www.consort-statement.org.
